# Circulating microRNAs Expression as Prognosis Biomarker of Cholangiocarcinoma

**DOI:** 10.31557/APJCP.2026.27.1.307

**Published:** 2026-01-21

**Authors:** Wanna Chaijaroenkul, Surawut Charoenkajonchai, Nisit Tongsiri, Kesara Na-Bangchang

**Affiliations:** 1 *Chulabhorn International College of Medicine, Thammasat University, Klong Luang, Pathumthani, 12120, Thailand.*; 2 *General Surgical Unit, Department of Surgery, Chonburi Hospital, Chonburi, Thailand.*; 3 *Sakon-Nakhon Regional Hospital, Sakon-Nakhon, Thailand.*

**Keywords:** Cholangiocarcinoma, miR-16-5p, miR-320e, miRNA, Nanostring Ncounter

## Abstract

**Background:**

Cholangiocarcinoma (CCA) is a significant problem in Southeast Asia, particularly Thailand. Changes in the expression of microRNAs (miRNA), is one of the mechanisms associated with the pathogenesis and progression of cancer.

**Objective:**

In the present study, serum miRNA expression from advanced-stage intrahepatic CCA patients was investigated using the high-throughput technique (Nanostring Ncounter© technology).

**Methods:**

Twenty-four intrahepatic CCA patients and eight healthy subjects were enrolled in this study. The CCA group was subgrouped according to disease progression into non-metastatic CCA and metastatic CCA.

**Results:**

Of the 803 miRNAs, expression of 239 miRNAs was significantly different among the three groups (p < 0.001). Among them, miR-302d-3p showed the most significant expression (p < 9.02x10^-7^, FDR: 7.25x10^-4^), with upregulation in patients with metastatic CCA compared to non-metastic CCA and healthy groups. Fold change analysis revealed that miR-320e expression was the most significantly upregulated across all three groups (p < 0.001). Additionally, the expression levels of miR-223-3p, miR-23a-3p, and miR-302d-3p were significantly increased in patients with both metastatic and non-metastatic CCA compared to healthy controls. Several miRNAs were significantly downregulated, among them, miR-16-5p and miR-451a showed significant downregulation in metastatic and non-metastatic CCA compared with healthy groups.

**Conclusions:**

These findings indicate that a panel of circulating miRNAs may serve as a useful tool for the diagnosis and prognosis of intrahepatic cholangiocarcinoma, warranting further validation in larger cohorts. Additionally, the accuracy of diagnostic tests may be improved by increasing the sample size and including diverse clinical subgroups to enhance the robustness and generalizability of the results.

## Introduction

Cholangiocarcinoma (CCA) is a highly invasive malignancy of the bile duct. The incidence rates of CCA vary significantly in different countries due to several factors, such as genetic differences and geographical variations in risk factors. CCA is a major cause of cancer death in northeastern Thailand [1], of which the significant risk factor is consumption of improperly cooked cyprinoid fish containing metacercariae of *Opisthorchis viverrini* (liver fluke) [1]. Chronic inflammation due to liver fluke induction causes to DNA damage in biliary epithelium cells, leading to abnormal cell proliferation and transformation. Several mechanisms are associated with the development and progression of CCA [2]. Apart from tumor pathogenesis, microRNAs (miRNAs), the small non-coding RNAs, are associated with tumor vascularization, invasion and the process of metastasis [3, 4]. It plays an essential role in post-translational regulation, which cleavages complementary mRNA, leading to gene repression [5]. For induction of mRNA degradation, miRNAs-mRNA interaction occurs at the 3’- untranslated region (3’ UTR) of target mRNAs, as well as other regions such as 5’ UTR, coding sequence, and gene promoters [6]. Several reports have confirmed the association between miRNAs and various types of cancer with regard to pathogenesis and biological processes, e.g., proliferation, differentiation, apoptosis, metabolism, invasion, metastasis, and drug resistance [6]. The localization of miRNA is both intracellular or extracellular. The extracellular miRNAs are present in the plasma/serum samples in a stable cell-free form [7]. 

Numerous reports indicate that abnormal miRNA expression may serve as a valuable biomarker for the diagnosis and prognosis of CCA [8]. Several oncogenic miRNAs, including miR-21, miR-221, miR-26a, miR-192, miR-877, miR-423-5p, miR-93-5p, and miR-4532 have been found to be upregulated in the serum or plasma of CCA patients [9-14]. Notably, the expression levels of miR-21 and miR-221 were significantly elevated in CCA associated with hepatolithiasis [9, 10]. A marked increase in serum miR-26a expression was correlated with metastatic CCA [11]. Elevated serum levels of miR-192, miR-423-5p, miR-93-5p, and miR-4532 were observed in patients with liver fluke-associated CCA [12, 14], while upregulated miR-877 showed potential as a biomarker for distal CCA [13]. Additionally, the combination of upregulated miR-877 and downregulated miR-16 demonstrated high sensitivity and specificity for the diagnosis of distal [13]. Other tumor-suppressor miRNAs, such as miR-150-5p, miR-106a, and miR-195, showed significant downregulation in the serum or plasma of CCA patients [15-17, 13].

Although their significance and clinical use remain unclear, the discovery of the new set of miRNAs promises new shed for CCA diagnosis, prognosis, and treatment. The present study investigated miRNA expression in Thai patients with CCA using high-throughput technique (Nanostring Ncounter© technology).

## Materials and Methods

### Study population

The study was conducted at Chonburi Hospital (Chonburi, Thailand) and Thammasat Chalermprakiet Hospital (Pathumtani, Thailand) during 2020 and 2022. The study protocol was approved by the Human Research Ethics Committee of Thammasat University, Faculty of Medicine (MTU-EC-OO-3-155/63) and the Human Ethics Committee of Chonburi Hospital, Chonburi province (133/63/O/h2). Blood samples were collected from stage-III and stage-IV CCA patients (aged 18-80 years) at Chonburi Hospital. The samples from healthy subjects were collected at Thammasat Chalermprakiet Hospital. Written informed consents were obtained from all subjects before the study.

### Sample processing

Clotted blood samples were centrifuged at 1,500 xg for 15 min, and serum samples were separated into two tubes. Biochemistry analysis included aspartate aminotransferase (AST), alanine transaminase (ALT), albumin, total protein, carbohydrate antigen 19-9 (CA 19-9) and carcinoembryonic antigen (CEA). The remaining serum samples were stored at -80ºC for molecular analysis.

### Extraction of miRNA

RNA was extracted from the serum samples using miRNeasy Kit (QIAGEN, Germany) according to the manufacturer’s protocol with modification. Briefly, 500 µl of serum was mixed with Qiazol reagent at the ratio of 1:5. The mixture was incubated at room temperature (25^o^C) for 5 minutes, and 500 µl of chloroform was added. The sample was thoroughly mixed and incubated at room temperature for 2-3 minutes, followed by centrifugation at 12,000 xg (4ºC) for 15 minutes. The supernatant was carefully transferred to a new tube, and 1.5x absolute ethanol was added. The mixture was transferred to the RNeasy mini-column and washed with Qiagen buffers according to the manufacture’s protocol. The miRNA was eluted with RNase-free water and stored at -80^O^C for further analysis. 

### Analysis of miRNA expression

The circulating miRNA expression panels were analyzed using Nanostring Ncounter© technology (NanoString Technologies, Seattle, WA, USA). This technology is a high-throughput, sensitive and reproducible method that can analyze the expression of more than 800 miRNA using molecular barcodes called nCounter Reporter Probes. The sample was annealed with a specific tag for each miRNA target using a color-coded probe pair (each probe consists of a Reporter Probe and a Capture Probe at 5′ and 3′ end, respectively). Target/probe complexes were bound to magnetic beads complementary to sequences on the capture probe. The capture probes and target/probe complexes were eluted off the beads and were hybridized into magnetic beads complementary to sequences on the reporter probe. Finally, the purified target/probe complexes were eluted off the beads and immobilized on the cartridge for data collection [18]. The selection of reference genes, as well as the positive and negative controls, was based on the manufacturer’s instructions. The complete protocol, including the list of genes and probes used in the assay, was provided by the manufacturer’s guidelines. The miRNA expression panel analysis (Human v3 miRNA Assay) was included 25 internal reference controls, as specified in the manufacturer’s catalogue (NanoString Technologies: https://nanostring.com/products/ncounter-assays-panels/immunology/mirna-expression-panels//)

### Data analysis

Data of the raw signals were submitted to quantile normalization. The miRNA expression data were analyzed by miRNet (https://www.mirnet.ca/). The fold-change analysis was performed by comparing the miRNA expression of serum samples from CCA patients and healthy subjects. The Heatmaps and Sparse Partial Least Squares - Discriminant Analysis (sPLS-DA) were used to identify differentially expressed miRNAs with the most predictive or discriminative features to classify the sample groups. The targets and their expression was explored via a public database (http://www.mirdb.org) to identify the possible targets (miRNA-targeted mRNAs). The Cytoscape software was used to visualize the miRNA–mRNA networks. All statistical significance was set at α = 0.05.

## Results

### Demographic data

Twenty-four CCA patients and eight healthy subjects were enrolled in this study (Table 1). All CCA patients were diagnosed with intrahepatic CCA (iCCA) and were categorized into two subgroups according to the disease progression, i.e., non-metastatic CCA (n=13) and metastatic CCA (n=11) subgroups. Other biochemical parameters, including AST, ALT, albumin, total protein, and CEA, did not differ significantly among the three groups. In contrast, serum CA19-9 levels were markedly elevated in patients with metastatic CCA compared to those with non-metastatic CCA (p <0.001) and there was also a statistically significant difference between CCA patients and healthy controls (p = 0.14).

### miRNA expressions in CCA patients and healthy subjects

**Figure 1:**
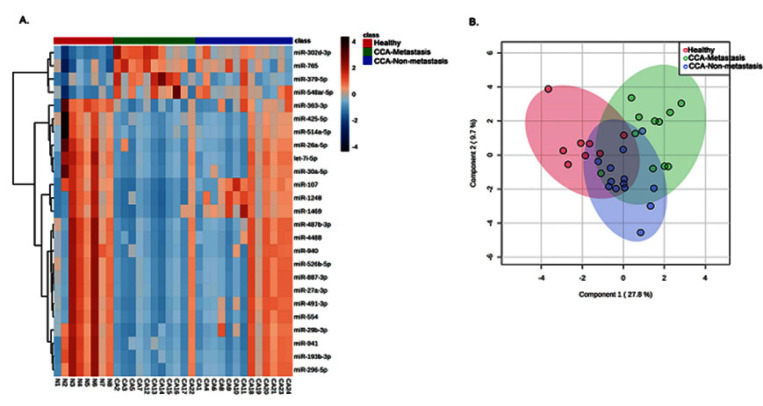
The Significant Expression of miRNA (A), (B). the expression of significant miRNA among the three groups, metastatic CCA patients (n=11), non-metastatic CCA patients (n=13), and healthy subjects (n=8).

The miRNA expression was analyzed in serum samples from metastatic CCA (n=11), non-metastatic CCA (n=13), and healthy subjects (n=8). Of the 803 miRNAs, the expression of 239 miRNA (Figure 1A) was significantly different among the three groups (p < 0.001). Among them, miR-302d-3p showed the most significant expression (p < 9.02x10^-7^, FDR: 7.25x10^-4^), with upregulation in patients with metastatic compared with non-metastic CCA and healthy groups.

**Figure 2.**
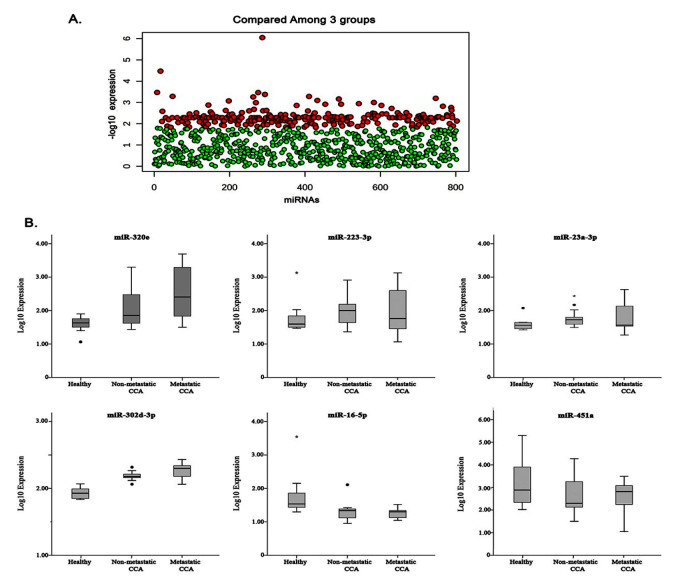
The Heatmaps of miRNAs Expression among three Groups: (A) the miRNAs with dark red were upregulated, and those with blue color were down-regulated, and (B) The 2D scores plot from sPLS-DA analysis.

For Heatmaps analysis (Figure 2A), the miRNAs were clustered, and differences among these three groups were determined. Four miRNAs (miR-302d-3p, miR-765, miR-379-5p, and miR-548ar-5p) were significantly upregulated in patients with metastatic CCA, compared with those with non-metastatic CCA and healthy subjects. The miRNAs expressions of the non-metastatic CCA patients and healthy subjects were similar in some subjects, while some showed a similar trend with metastatic CCA.

For further discrimination of miRNA expression data among these three groups, the sparse PLS-DA (sPLS-DA) was analyzed and results are shown in Figure 2B. Ten dominant miRNAs, i.e., miR-302d-3p, let-7i-5p, miR-29b-3p, miR-30a-5p, miR-107, miR-26a-5p, miR-487b-3p, miR-765, miR-193b-3p, and miR-526b-5p showed 27.8% of the variance in the data matrix. Of them, miR-302d-3p and miR-765 were found to be upregulated, which was also identified in the Heatmaps analysis.

### Fold-change analysis

The fold-change (FC) analysis of miRNAs expression (upregulation and downregulation) in serum samples from all groups of subjects is shown in Table 2. miR-320e was found to be significantly upregulated in all groups. In patients with metastatic CCA, 11 miRNAs (miR-320e, miR-223-3p, miR-122-5p, miR-23a-3p, miR-302d-3p, miR-4516, miR-379-5p, miR-548ar-5p, miR-1246, miR-548n, and miR-765) were upregulated. The expression of miR-320e showed the highest fold-change (logFC = 5.28). In non-metastatic CCA patients, 4 miRNAs (miR-320e, miR-223-3p, miR-23a-3p, miR-302d-3p) were significantly upregulated. These miRNA were the subgroup of the metastatic group, but the degree of expression was different. When comparing between metastatic CCA and non-metastatic CCA, only miR-320e showed significant upregulation, while 2 miRNAs (miR-16-5p and miR-451a) were significantly downregulated (Figure 1B).

### miRNA–mRNA network

**Figure 3:**
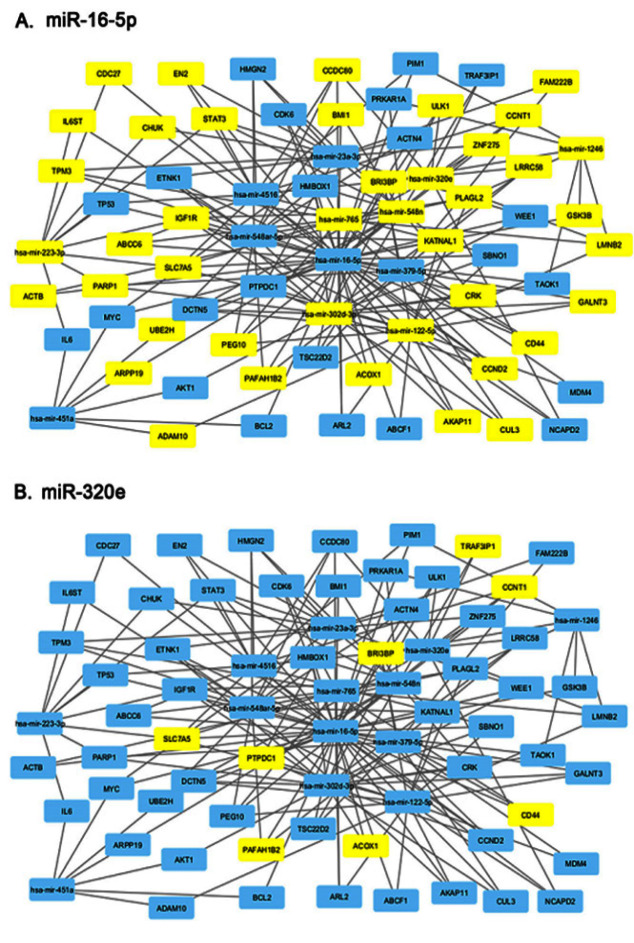
miRNA-mRNA Network. A. the selected node (yellow color) linked with miR-16-5p. B. the selected node (yellow color) linked with miR-320e.

The enrichment analysis and KEGG pathway analysis on the target mRNAs were performed using miRNet to explore the potential function of significant mRNAs. The miRNA-mRNA network was constructed with 13 significant miRNAs (Table 3). As shown in Figure 3, the related pathways are involved in cancer-related pathways (KEGG:05200; p-value = 6.72E-08, FDR = 5.71E-06), cell cycle (KEGG: 04110 ; p-value = 5.77E-06, FDR = 0.000163), focal adhesion (KEGG:04510; p-value = 1.44E-05, FDR = 0.000204), Jak-STAT signaling pathway (KEGG:04630; p-value = 1.99E-05, FDR = 0.000211), apoptosis (KEGG:04210; p-value = 0.000111, FDR = 0.000944), and p53 signaling pathway (KEGG:04115; p-value = 0.000642, FDR = 0.00385). This miRNA network may be linked with the regulation of CCA cell proliferation, migration, and invasion. 

The gene enrichment analysis revealed that miR-16-5p is the central node that connects with GSK3B, CCNT1, mir-320e, BMI1, ACTB, LMNB2, IGF1R, CCND2, AKAP11, PEG10, CDC27, mir-1246, CHUK, PARP1, TPM3, ABCC6, EN2, ADAM10, SLC7A5, FAM222B, CCDC80, KATNAL1, ACOX1, ULK1, IL6ST, mir-223-3p, CRK, CD44, PAFAH1B2, LRRC58, ZNF275, mir-122-5p, CUL3, ARPP19, mir-548n, mir-765, PLAGL2, UBE2H, GALNT3, STAT3, mir-302d-3p, and BRI3BP (Figure 3A, p-value =3.35E-26). The miR-320e has been linked with CCNT1, TRAF3IP1, PTPDC1, SLC7A5, ACOX1, CD44, PAFAH1B2, and BRI3BP (Figure 3B, p-value = 4.42E-08).

### miRNA target search: genes and proteins

The miRNA-320e was selected for further target search using mirdb.org database (http://www.mirdb.org/index.html) as it was significantly upregulated among the three groups (Table 2). The highest target score with high expression in the four CCA cell lines (HuCCT1, SNU-1079, SNU-1196, and SNU-245) was selected as possible targets and the major target genes identified were LAPTM4A and STT3B (Table 4). These two genes have been predicted to link with several proteins (https://string-db.org).

**Figure 4:**
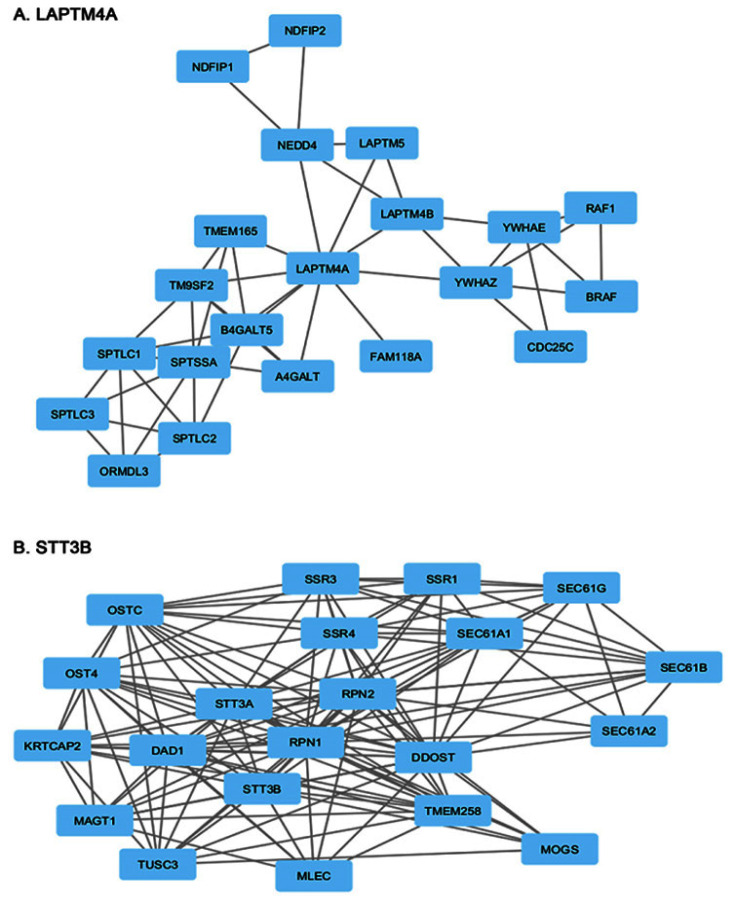
Protein Network Predicted from String Database for (A) LAPTM4A and (B) STT3B.

The enrichment of KEGG predicted the link of LAPTM4A gene with the sphingolipid signaling pathway (KEGG:04071, p-value = 1.13E-04, FDR=0.0024) and sphingolipid metabolism (KEGG:00600, p-value= 1.31E-04, FDR = 0.0033) (Figure 4A). The STT3B gene was predicted to be linked with protein processing in the endoplasmic reticulum (KEGG:04141, p-value = 1.72E-24, FDR = 4.83E-22) and N-Glycan biosynthesis (KEGG:00510, p-value = 4.40E-14, FDR = 9.69E-14) (Figure 4B).

## Discussion

miRNAs play a major role in tumor progression by regulating target genes involved in tumor proliferation and metastasis [19]. Several miRNAs have been reported in CCA patients’ tissues and serum/blood [10, 20]. The present study showed significant differences in the expression profiles of miRNAs among the three groups. The most promising candidate miRNAs that could differentiate patients with metastatic CCA from those with non-metastatic CCA and healthy subjects were miR-320e, miR-223-3p, miR-23a-3p, miR-302d-3p, miR16-5p, and miR-451a.

The tumor-suppressive role of the miR-320 family (miR-320a, b, c, and d) has been reported in various cancer types [21-23]. Its expression was upregulated in patients with colorectal cancer who had recurrence compared with those without recurrence. In addition, it was found to be upregulated in esophageal adenocarcinoma compared with Barrett’s esophagus [24, 25]. However, the role of miR-320e in cholangiocarcinoma (CCA) remains unclear. One study reported low expression of miR-320 in CCA tissues, though the specific family member was not identified [26]. Consistent with this, the present study demonstrated significant upregulation of miR-320e in patients with metastatic CCA compared to those with non-metastatic CCA and healthy controls. While the function of miR-320e is not yet fully understood, it is predicted to function as a post-transcriptional regulator of gene expression, potentially contributing to various cellular processes, including osteogenesis, metabolic regulation, and cancer suppression by modulating cell proliferation and apoptosis [27, 28].

The current results showed overexpression of miR-223 in CCA patients, which has also been reported in several other cancers, including gastric, hepatocellular, ovarian, lung, and esophageal cancers [29-32]. miR-223 plays key roles in regulating apoptosis, as well as cell proliferation, migration, and invasion. High expression of miR-223 has been linked to colorectal [33] and ovarian cancers [34]. However, miR-223 downregulation was reported with high tumor burden, disease aggressiveness, and poor patient prognosis in some cancers [35, 36]. Additionally, overexpression of miR-223-3p in oral squamous cell carcinoma has been implicated in cisplatin resistance [37]. Therefore, the role of miR-223 appears to be either oncogenic or tumor-suppressive functions depending on the cancer type.

The expression of cancer-promoting gene, miR-23a-3p, was reported in several cancers, including pancreatic cancer and malignant melanoma [38-40], and CCA [41]. The miR-23a-3p overexpression in CCA tissues and cell lines being associated with increased cell proliferation, invasion, and metastasis. Inhibition of miR-23a-3p prevented cancer cell proliferation both in vitro and in vivo. This miRNA is secreted from CCA cells via exosomes. In agreement with that previously reported in CCA cell line and tissues [41], overexpression of miR-23a-3p was found in serum samples from CCA patients in the current study. 

The miR-302d-3p is a member of the miR-302 family. Its role in CCA is not clearly understood. However, theis miR-302d-3p is predicted to regulate the viability, migration and apoptosis of cancers [38]. Our results indicated that miR-302d-3p expression was upregulated depending on the status/progression of CCA. The expression was also highly upregulated in patients with metastatic CCA compared with non-metastatic. This miRNA was shown to be downregulated in human glioblastoma and breast cancer cells [42-44]. On the other hand, it was shown to be upregulated in tumor tissues of patients with hepatocellular carcinoma (HCC), and patients with high expression of miR-302d had shorter survival time than those with low expression [45]. Thus, miR-302d expression in cancer varies depending on the type of cancer. Correlation between miRNA expression of tissue and serum samples has also been demonstrated [46, 47]. 

The miR-16-5p was downregulated in serum samples from CCA patients in this study. Previous studies also reported the downregulation in cancer cell lines and clinical samples [48]. Downregulation of miR-16-5p was associated with the development of diverse malignancies, including neuroblastoma, osteosarcoma, hepatocellular carcinoma, cervical cancer, breast cancer, brain tumor, gastrointestinal cancer, lung cancer, and bladder cancer [49-57]. In CCA, the expression of miR-16-5p was low in CCA tissues, and correlated with tumor size, tumor metastasis, and TNM stage [48]. Both in vitro and in vivo studies demonstrated that miR-16 could suppress proliferation, invasion and metastasis throughout the progression of CCA, which was associated with poor survival in CCA patients [58]. The suppression of miR-16-5p increased YAP1 expression, and thus, promoting CCA proliferation [59]. Moreover, miR-16-5p has also been associated with chemosensitivity, radiosensitivity, and response to the targeted therapy.

The miR-451 plays a role in multiple physiological and pathological processes, including hematopoietic system differentiation, embryonic development, epithelial cell polarity, and nervous system development [3, 60, 61]. Downregulation of miR-451 was reported in several types of cancer [62-64]. Guo et al. [65] reported the downregulation of miR-451 in tissue and serum samples from CCA patients, which were associated with the progressive disease, i.e., TNM Classification of Malignant Tumors (TNM) stage and positive lymph node metastasis. Thus, the miR-451a could be a promising tumor suppressor in CCA.

The study identified the key node (mRNA) linked to a significant miRNA could predict miRNA-mRNA network, which further predicted the functions of significant miRNAs. The two major miRNAs, i.e., miR-16-5p and miR-320e, might be useful for the prognosis markers of CCA. The miR-16-5p has been linked with GSK3B, which acts as either a proapoptotic and an antiapoptotic factor [66]. GSK3B has been reported to be associated with the cytotoxic activity of doxorubicin in human CCA cells. 

Two possible targets of miR-320e, i.e., LAPTM4A and STT3B, were identified. Both have been associated with sphingolipid and protein processing in the endoplasmic reticulum/N-Glycan biosynthesis, respectively. Sphingolipilid is crucial in regulating tumor progression in response to anticancer therapy [67]. Endoplasmic reticulum stress has been related to recurrence, metastasis, and drug resistance in cancers [68, 69]. The extended high-mannose glycans have been reported to be more abundantly expressed in metastatic CCA, which is supported by the predicted function of miR-320e that highly expresses protein regulating N-Glycan biosynthesis in metastatic CCA patients [70]. 

This study has several limitations. First, the sample size was relatively small, which may limit the statistical power and generalizability of the findings. Second, all serum samples were collected from patients within a single geographic region, potentially introducing regional bias. Third, the identified miRNA biomarkers were not validated in corresponding tissue samples, which limits the understanding of their biological relevance and source. Future studies with larger, multi-center cohorts and tissue-based validation are needed to strengthen these preliminary findings.

**Table 1:** The Demographic data of CCA and Healthy Subjects. Qualitative data are presented as number (%) and quantitative data are presented as median (range) values.

	CCA (n=24)	Healthy (n=8)
Gender		
Female	12 (50%)	4 (50%)
Male	12 (50%)	4 (50%)
Age (years)		
< 60	10 (42%)	7 (88%)
> 60	14 (58%)	1 (12%)
Disease Stage		-
Stable	13 (54%)	
Metastasis	11 (46%)	
CEA (ng/mL)	3.6 (0.5-29.4)	1.2 (0.5-3.2)
	Non-metastatic: 2.9 (0.5-17.6)	
	Metastatic: 3.7 (1.0-29.4)	
CA19-9 (U/mL)	273.0 (1.2-6702.5)	11.2 (2.4-39.7)**
	Non-metastatic: 9.3 (1.2-15.5)	
	Metastatic: 2790.2 (39.3-6702.5)*	
ALT (U/L)	30.0 (7.0-81.0)	28.0 (17.0-46.0)
	Non-metastatic: 24.0 (15.0-33.0)	
	Metastatic: 33.0 (7.0-81.0)	
AST (U/L)	51.0 (21.0-151.0)	22.0 (14.0-31.0)
	Non-metastatic: 27.0 (21.0-151.0)	
	Metastatic: 52.0 (24.0-100.0)	
Albumin (g/dL)	3.1 (1.8-4.4)	4.2 (3.5-4.4)
	Non-metastatic: 3.3 (1.8-4.3)	
	Metastatic: 2.9 (2.2-4.4)	
Total protein (g/dL)	7.9 (6.0-8.8)	8.1 (7.1-8.6)
	Non-metastatic: 7.9 (6.9-8.5)	
	Metastatic: 33.0 (6.0-8.8)	

* Statistically significant between CCA constant and metastasis (p <0.001); ** Statistically significant between CCA and healthy (p = 0.014)

**Table 2:** The Fold-Change (FC) Analysis of the Significant miRNA in Patients with Metastatic CCA, Non-Metastatic CCA, and Health Subjects (Fold-Change Cut off >2.0 and <0.5).

MiRNAs	Metastatic CCA/ Healthy		Non-metastatic CCA/ Healthy		Metastatic CCA/ Non-metastatic CCA	
	Log2FC (95%CI)	P-value	Log2FC (95%CI)	P-value	Log2FC (95%CI)	P-value
miR-320e	5.28	9.80E-34	3.28	3.94E-17	2	7.21E-11
	(2.47-5.28)		(-2.41-3.47)		(1.82-4.88)	
miR-223-3p	2.57	1.10E-11	1.49	2.59E-05	NS	NS
	(1.96-3.70)		(-0.32-2.80)			
miR-122-5p	2.2	8.01E-09	NS	NS	NS	NS
	(2.00-2.83)					
miR-23a-3p	2.13	1.81E-08	1.42	7.99E-05	NS	NS
	(1.74-5.75)		(-2.41-3.91)			
miR-302d-3p	1.78	1.20E-06	1.31	1.92E-04	NS	NS
	(0.96-1.89)		(0.82-1.63)			
miR-4516	1.75	3.35E-06	NS	NS	NS	NS
	(0.90-1.82)					
miR-379-5p	1.48	7.57E-05	NS	NS	NS	NS
	(1.00-1.62)					
miR-548ar-5p	1.34	2.34E-04	NS	NS	NS	NS
	(0.96-1.56)					
miR-1246	1.31	3.10E-04	NS	NS	NS	NS
	(0.82-2.16)					
miR-548n	1.31	4.23E-04	NS	NS	NS	NS
	(-0.04-1.92)					
miR-765	1.25	5.10E-04	NS	NS	NS	NS
	(1.05-1.41)					
miR-16-5p	-1.63	3.91E-06	-1.53	4.63E-06	NS	NS
	(-5.01 - -1.32)		(-5.79 - -1.24)			
miR-451a	-2.1	8.38E-10	-1.21	1.81E-04	NS	NS
	(-6.70 - -2.03)		(-5.43 - -1.06)			

*NS, not significant

**Table 3:** The KEGG Function Predicted from miRNA-mRNA Network

Pathway	Total	Expected	Hits	P-value	FDR
Pathways in cancer	310	1.85	12	6.72E-08	5.71E-06
Cell cycle	124	0.742	7	5.77E-06	1.63E-04
Focal adhesion	200	1.2	8	1.44E-05	2.04E-04
Jak-STAT signaling pathway	99	0.592	6	1.99E-05	2.11E-04
Apoptosis	83	0.497	5	1.11E-04	9.44E-04
p53 signaling pathway	68	0.407	4	6.42E-04	3.85E-03

**Table 4:** The Predicted Target for miR-320e

Target Rank	Target Score	Gene Symbol	Expression in CCA cells				Gene Description
			Hu	SNU-	SNU-	SNU-	
			CCT1	1079	1196	245	
1	97	USP48	6	5	5	6	Ubiquitin-specific peptidase 48
2	96	SLC10A3	21	9	17	19	Solute carrier family 10 member 3
3	96	SKA3	14	4	11	13	Spindle and kinetochore-associated complex subunit 3
4	95	STT3B	54	37	32	50	STT3B, the catalytic subunit of the oligosaccharyltransferase complex
5	95	TMEM47	4	-	-	-	Transmembrane protein 47
6	95	LAPTM4A	98	130	82	100	Lysosomal protein transmembrane 4 alpha
7	94	TSHZ3	-	-	-	-	Teashirt zinc finger homeobox 3
8	94	VEGFD	-	-	-	-	Vascular endothelial growth factor D
9	92	FBXO45	7	3	5	6	F-box protein 45
10	92	PNRC1	10	7	2	4	Proline-rich nuclear receptor coactivator 1

## Author Contribution Statement

Wanna Chaijaroenkul carried out the experiments, data analysis and draft manuscript. Surawut Charoenkajonchai and Nisit Tongsiri were involving planning and supervised the work. Kesara Na-Bangchang participated in study design and data interpretation. All authors provided critical discussion on results and contributed to the final manuscript. 
